# The Relationship between Social Media Use and Body Image in Lebanese University Students

**DOI:** 10.3390/nu15183961

**Published:** 2023-09-13

**Authors:** Joanne M. Karam, Carol Bouteen, Yara Mahmoud, Josep A. Tur, Cristina Bouzas

**Affiliations:** 1 Institut National de Santé Publique, d’Épidémiologie Clinique et de Toxicologie-Liban (INSPECT-LB), Beirut 1103 2180, Lebanon; 2 Department of Natural Sciences, Lebanese American University, Beirut 1102 2801, Lebanon; 3 School of Health Sciences, Modern University for Business and Science, Damour 113-7501, Lebanon; 4 Research Group on Community Nutrition and Oxidative Stress, University of the Balearic Islands-IUNICS, E-07122 Palma de Mallorca, Spain; cristina.bouzas@uib.es; 5 Health Research Institute of Balearic Islands (IdISBa), E-07120 Palma, Spain; 6 CIBEROBN (Physiopathology of Obesity and Nutrition CB12/03/30038), Instituto de Salud Carlos III, E-28029 Madrid, Spain

**Keywords:** body image, self-perception, social media addiction, social media use, university students, Lebanon

## Abstract

Well-being is not only defined as being physically healthy; multiple factors can affect a person’s well-being. Social media is strongly correlated with the body dissatisfaction of an individual. High exposure to lean and toned body shapes has created new standards and “idealized” body types. The aim of this article was to assess the relationship between social media and body image among university students in Lebanon. Data were obtained from 292 university students (median age: 22 years), selected from different Lebanese regions by using convenience sampling. Demographic data, social media addiction, body satisfaction, levels of physical activity, eating behaviors, and ultimate well-being were expressed as median and interquartile range. People who relied more on social media were younger than those who did not. Individuals addicted to social media had higher odds of having moderate and marked body image concerns. A significant association was found between social media addiction and emotional overeating, food responsiveness, and feeling hunger. These findings stress the need for rising regional and national awareness among social media users, especially the younger ones, and the implementation of intervention and prevention techniques to help prevent body image dissatisfaction, disordered eating patterns, and the alteration of overall well-being.

## 1. Introduction

The World Health Organization (WHO) defines health as a “state of complete physical, mental, and social well-being” and not merely the absence of disease or infirmity [1]. Human beings seek to feel well in their lives, which makes well-being a vital human aim [2]. The concept of well-being describes how people feel about their lives, which may or may not be strongly correlated with the unchanging realities of people’s lives [3]. According to the center for disease control, CDC, well-being is associated with job, family, health, and economically related benefits [2]. Psychological well-being specifically is a main building block of mental health and may be defined as enjoyment and pleasure on one hand and meaning and fulfillment on the other hand, as well as resilience (coping, emotional regulation, and healthy problem solving) [4].

Among university students, well-being is specifically important as it is a critical contributor to lifelong fulfilment [5], but it is at risk since the students encounter many academic difficulties, as well as social, emotional, and psychological ones [6].

Lebanon is undergoing several crises arising due to a massive economic collapse and the tragic August 4 Beirut port blast [7]. Young adults representing 28% of the total population were found to be most affected by these crises because they are the population most vulnerable to stressors since their future is at stake, and it is their time to shape their lives in a country that is crippling them [8].

Social media has become the primary source of communication for young people, and its usage has increased substantially among university students, who mainly use it for non-academic purposes [9]. It presents significant challenges for the younger generation since it allows them to discover the world as a filtered image rather than portraying the real world [10]. Social media is responsible for the new perception of what is called an “ideal man” or “ideal woman”. Those terms refer to men or women socially perceived as attractive, according to sociocultural norms [11]. Moreover, this is considered the primary cause of the exertion of extra pressure on the public, which leads to body dissatisfaction due to the unattainable standards that are set in society [12]. The social comparison theory might explain this association as it asserts that individuals tend to compare themselves to others who are similar [13]. This theory posits that individuals evaluate themselves by comparing their abilities and opinions to those of others, which influences their self-perception and emotional well-being [14].

People frequently display an idealized picture of themselves on social media, adding only the most appealing photographs of themselves to their profile [15]; those pictures might be even changed using applications and filters. Even though social media provides images of a variety of individuals (e.g., friends, family, strangers, and celebrities), it is mainly used to communicate with one’s peers [16].

Evidence shows that peer comparisons may have a particularly strong impact on body image [17]. Physique dissatisfaction is frequently tested by asking people to rate their actual body with their ideal form; as with social comparison theory, the gap between the two is the level of body dissatisfaction [12]. Body image and psychological well-being are inextricably linked; body dissatisfaction is mainly based on a person’s negative thoughts about their appearance, which are frequently influenced by social experiences like media representations [18]. In recent years, body dissatisfaction has become a serious worry for both men and women, particularly women [19,20]. Body dissatisfaction has previously been related to unhealthy lifestyles, whereas body satisfaction has been related to healthier lifestyles [20]. Therefore, authors hypothesize that the usage of social media could be linked to body dissatisfaction and to lifestyle.

Understanding and investigating students’ body dissatisfaction as influenced by the effects of social media is significantly important in assessing the need to develop appropriate education on health awareness and the appropriate use of social media. Such studies are lacking, especially among university students in Lebanon during the crisis. This study aims to assess the relationship between social media and body image among university students in Lebanon.

## 2. Methods

### 2.1. Design and Sample Size

This study is a cross-sectional survey. Participants were asked to complete an online questionnaire including six sections. This survey was designed to assess the effect of social media on body image. For data collection purposes, the online survey was distributed throughout different social media platforms. The inclusion criterion for subjects to participate in this study consisted of being Lebanese university students registered in different universities across all Lebanese regions. Exclusion criteria included being a Lebanese student outside Lebanon and being a non-Lebanese student in a university in Lebanon. The subject population included both genders regardless of the educational level or occupation of the participants. A convenience sampling technique was used in this research. The sample size was 292 participants. Data collection took place in November and December 2021.

This study received approval from the ethical committee of the Modern University of Business and Science (approval reference MU-20211105-27). The survey included a consent form. Subjects had the full right to drop out of the study at any time they wanted without giving any reason. Participants were asked to complete a 10 min questionnaire. All questionnaire responses were confidential and anonymous, and the analyzed data were non-identifiable.

### 2.2. Assessment Tools

The questionnaire, distributed in English, included 6 sections: (1) sociodemographic. (2) The Bergen social media usage scale (BSMAS), a six-item scale used to assess addiction to social media [21], from which scores were calculated according to a certain ranking (any level above 18 was considered an addiction to social media). In the present study, the internal consistency of BSMAS was quite acceptable (Cronbach α = 0.762). (3) The Body Shape Questionnaire 16-Item Version (BSQ-16), a self-reported measurement of body shape concerns [22]; scores were also recorded and classified in a system split into four categories: ‘no concern’ (scoring less than 38), ‘mild concern’ (scoring between 38 and 51), ‘moderate concern’ (scoring between 52 and 66), and ‘marked concern with shape’ (scoring over 66) (Cronbach α = 0.945). (4) The Godin leisure scale, a four-item scale used to assess physical activity [23], in which scoring was as follows: active (scoring 24 units or more), moderately active (scoring between 14 and 23 units), and insufficiently active/sedentary (less than 14 units), and “how often they work up a sweat”, to which the possible answers were often, sometimes, and never (Cronbach α = 0.649). (5) The Adult Eating Behavior Questionnaire (AEBQ), used to assess positive and negative beliefs about food and eating [24] and which consists of 35 items, was rated on a 5-point Likert-type scale (1–strongly disagree; 5–strongly agree); these items were split into sub-categories which are as follows: hunger, food responsiveness, emotional eating, enjoyment of food, satiety responsiveness, emotional undereating, food fussiness, and slow eating (Cronbach α for each sub category = 0.700–0.895). (6) The last part of the questionnaire was the World Health Organization-5 Well-being Index (WHO-5) validated in Lebanon and used to assess psychological well-being [25,26], which consisted of five statements, rated according to the following scale: all of the time 5, most of the time 4, more than half of the time 3, less than half of the time 2, some of the time 1, at no time 0 (Cronbach α = 0.843). The total raw score, ranging from 0 to 25, was multiplied by 4 to give the final score, with 0 representing the worst imaginable well-being and 100 representing the best imaginable well-being. All variables were calculated as the authors of the questionnaire and the validation study have stated.

Addiction status was classified according to the total score of BSMAS. The median value was 18 points; therefore, a total score > 18 was an addiction indicator, while a total score ≤ 18 was a no-addiction indicator. All variables from BSQ-16, weekly leisure-time activity, and WHO-5 that were not dichotomous were transformed considering each specific study issue as a cutoff point. All variables from AEBQ that were not dichotomous were transformed taking the median as a cutoff point.

### 2.3. Statistics

The SPSS statistical software package version 27.0 (SPSSS Inc., Chicago, IL, USA) was used to perform analyses. The distribution of variables was assessed by performing the Kolmogorov–Smirnov test. The data are shown as median and interquartile range (IQR), and differences among groups were analyzed by performing the Mann–Whitney U test because the variables did not follow a normal distribution. The prevalence is shown as sample size and percentage. The difference in prevalence among groups was analyzed using χ^2^ (all *p* values are two-tailed). The association between body shape concern, physical activity level, well-being status, and adult eating behaviors (dependent variables) and addiction status (independent variables) was analyzed by calculating the odds ratio (OR). For each item, two ORs were calculated: crude and adjusted by sociodemographic characteristics (age, gender, and university student). Results were considered statistically significant when the *p*-value < 0.05.

## 3. Results

Sociodemographic characteristics according to social media addiction (no addiction: total BSMAS score ≤ 18; addiction: total BSMAS score > 18) are shown in Table 1. Participants with a major social media addiction were slightly younger (21 years) than those who did not have an addiction.

Body shape concern, weekly leisure-time activity, and well-being index according to social media addiction are available in Table 2. Most people without social media addiction are not concerned with their body shape (63.9%). In the social media addiction group, the percentage of participants who had moderate and marked concern with their body shape was notably high (31.2%).

Eating behaviors according to social media addiction are shown in Table 3. Regarding the enjoyment of food, participants obtained the same score in both groups (4.0). Nevertheless, the scores referring to emotional overeating, food responsiveness, and hunger were higher in the social media addiction group participants, at (3.2)/(3.3)/(3.4), respectively.

In Table 4, crude and adjusted ORs were calculated to find an association between body shape concern, weekly leisure-time activity, well-being index, and social media addiction. No addiction was established as the reference. Crude and adjusted analysis showed that students addicted to social media had higher odds of reporting moderate and marked concern with shape (adjusted OR: 0.48; 95% CI: 0.30–0.77). Accordingly, students with social media addiction were less likely to report no concerns with body shape than students without addiction (adjusted OR: 3.01; 95% CI: 1.70–5.33).

Table 5 shows crude and adjusted ORs for association between eating behaviors and social media addiction. No addiction was established as the reference (1.00). In all cases, crude and adjusted analyses showed that the OR for the addiction group was higher than for the no-addiction group for “Emotional overeating” (adjusted OR: 2.2; 95% CI: 1.42–3.54), “Food responsiveness” (adjusted OR: 2.13; 95% CI: 1.35–3.36), and “Hunger” (adjusted OR: 2.16; 95% CI: 1.37–3.41) items. Hence, students with social media addiction were more likely to report emotional overeating, food responsiveness, and hunger than students without social media addiction.

## 4. Discussion

The current study explored the effect of social media on body image among university students in Lebanon. Younger participants displayed major social media addiction compared to older participants. Social media addiction was also more dominant among young women (80.5%) than among men. Similar findings were obtained in Turkey [27] and Romania [28]. These results are also consistent with prior reports of social media addiction, where findings showed that young people [29,30,31,32,33,34,35] and females [36] were more prone to social media addiction. The tendency of the young population to be addicted to social media can be attributed to their need to express their personality, achieve dominance, escape family pressure, overcome loneliness, and earn social approval, in addition to coping with psychological disorders, economic problems, and physical inabilities [27]. Negative mood states are also considered causes of social media addiction among young people; these include timidity, depression, social phobia, and worry about the future. Hence, a strong body of evidence relates youth and social media addiction. However, other studies have shown significant relationships between males and older adults with problematic social media use or who have not displayed any relationship [37,38,39,40], which reflects that there is no agreement among studies on the association of a particular sociodemographic with social media addiction.

A positive association between no addiction to social media and no concerns about body shape was determined in this current study. Moreover, the percentage of participants having moderate and marked concerns with body shape was significantly higher among the social media addiction group compared to the group not addicted to social media. Previous studies examining the relationship between social media addiction and body image concerns yielded comparable results [41,42], especially among females [12,43,44,45].

As has been pointed out [46], the digital filters that hide flaws set an unrealistic standard of beauty. Moreover, studies have shown that internalizing the portrayed images exposed by the media leads to a desire to achieve ideal beauty [47,48]. This association can be explained by the social comparison theory, suggesting that individuals tend to compare themselves to others who are similar to them [13]. In fact, the significant Western influence in the media in Lebanon has spread perceptions of a ‘perfect body’ and thus led to increased body concerns [45]. However, these results contradict other studies that did not find a direct association between social media addiction and body concerns or dissatisfaction [49,50,51,52].

The social media addiction group obtained higher scores for emotional overeating, food responsiveness, and hunger. These current results confirm previous studies among Lebanese university students, where eating disorders (mainly emotional and restrained eating) were significantly linked to social media influence and pressure [53], and separation anxiety from technological devices was associated with the risk of eating disorders [54]. Similar findings were obtained in a recent study evaluating the relationship between social media addiction and eating behavior and disorder risk in university students during the pandemic period in Turkey, where emotional eating behavior was higher among social media addiction participants [55]. This was also supported by the literature on the impact of social media addiction on eating behaviors [56,57,58,59]. Research in America has also shown that social media use can affect eating choices and increase individuals’ unaware food intake despite their not feeling hungry [60]. In fact, the use of social media can influence eating choices and food intake due to product marketing and excessive energy-dense food exposure [61], in addition to peer pressure, as young adults tend to pay increased attention to products recommended by friends on social media [62]. Emotional eating can also result in overeating [63] and higher energy-dense food intake in attempts to rapidly reduce negative moods [64].

### Strengths and Limitations

The current study addresses a currently hot topic that affects health and well-being. Moreover, it addresses students, who are at most risk of developing social media addiction, specifically in the context of Lebanon, where it might become an escape mechanism due to the escalating humanitarian crisis [7], with no previous studies having tackled this subject since the crisis boomed in 2019. Tools used for the current research were previously validated, providing the article with scientific soundness. Nevertheless, this study also has limitations. It was conducted using online self-reported data, which introduces a risk of memory bias. Moreover, the studied population is limited to Lebanese university students, which might be a hindrance in extending the findings to other populations. In addition, not all the instruments used were previously validated in Lebanon. Last but not least, due to the cross-sectional nature of the study, there is a potential reverse causation, as its cross-sectional design does not allow it to explore whether the behaviors are an outcome or an antecedent of social media use [65]. Therefore, the use of social media and body concerns or behaviors could have an inherent relationship that cannot be ascertained due to the study’s design.

## 5. Conclusions

Social media addiction among Lebanese students is related to body image concerns and several behaviors/feelings such as emotional overeating, food responsiveness, and feeling hunger. Such associations increase the risk of unhealthy behaviors, especially those related to food intake, which will directly affect health.

These findings should raise awareness among social media users, especially younger users, of the need to avoid the detrimental effects on health of social media overuse. These findings should be taken into consideration by authorities regulating social media access and when developing programs to address eating behavior disorders.

Longitudinal research would be advisable to be able to better establish causality and to check possible interventions to improve such associations.

## Figures and Tables

**Table 1:** Sociodemographic characteristics of participants according to social media addiction.

	No Addiction (*n* = 166)	Addiction (*n* = 154)	*p*-Value
Age (years) *	22.0 (8.0)	21.0 (4.0)	0.017
Gender (female; n; %)	138 (83.1)	124 (80.5)	0.544
Province (n; %)			0.916
Beirut	17 (10.3)	16 (10.5)	
Mount Lebanon	119 (72.1)	108 (70.6)	
North of Lebanon	2 (1.2)	1 (0.7)	
South of Lebanon	10 (6.1)	10 (6.5)	
Begaa	16 (9.7)	15 (9.8)	
Nabatiyeh	1 (0.6)	3 (2.0)	

* Values are median (IQR: interquartile range). Differences between groups were tested by performing the Mann–Whitney U test. Differences in prevalence across groups were examined using χ^2^.

**Table 2:** Body shape concern, weekly leisure-time activity, and well-being index according to social media addiction.

	No Addiction	Addiction	*p*-Value
	(*n* = 166)	(*n* = 154)	
BSQ-16 (n; %)			0.001
No concern with shape	106 (63.9)	74 (48.1)	
Mild concern with shape	37 (22.3)	32 (20.8)	
Moderate and marked concern with shape	23 (13.9)	48 (31.2)	
Weekly Leisure-Time Activity (n; %)			0.563
Insufficiently Active/Sedentary	67 (40.4)	55 (35.7)	
Moderately Active	24 (14.5)	28 (18.2)	
Active	75 (45.2)	71 (46.1)	
WHO-5 (n; %)			0.137
Worst possible well-being	2 (1.2)	6 (3.9)	
Poor well-being	44 (26.5)	51 (33.1)	
Mild well-being	66 (39.8)	60 (39.0)	
Good and best possible well-being	54 (32.5)	37 (24.0)	

Abbreviations: BSQ-16: Body Shape Questionnaire 16-Item Version; WHO-5: World Health Organization-5 Well-being Index. Differences in prevalence were examined using χ^2^.

**Table 3:** Eating behaviors according to social media addiction.

	No Addiction	Addiction	*p*-Value
	(*n* = 166)	(*n* = 154)	
AEBQ *			
Enjoyment of food (EF)	4.0 (1.0)	4.0 (1.0)	0.028
Emotional overeating (EOE)	2.6 (1.4)	3.2 (1.6)	<0.001
Emotional undereating (EUE)	3.2 (1.4)	3.0 (1.6)	0.409
Food fussiness (FF)	2.2 (1.0)	2.4 (1.1)	0.245
Food responsiveness (FR)	3.0 (1.0)	3.3 (0.8)	<0.001
Hunger (H)	3.0 (0.8)	3.4 (1.0)	<0.001
Slowness in eating (SE)	2.8 (1.3)	2.8 (1.5)	0.705
Satiety responsiveness (SR)	2.8 (1.0)	3.0 (1.2)	0.067

* Values are median (IQR: interquartile range). Abbreviation: AEBQ: Adult Eating Behavior Questionnaire. Differences between groups were tested by performing the Mann–Whitney U test.

**Table 4:** Association between body shape concern, weekly leisure-time activity, and well-being index and social media addiction.

		No Addiction (*n* = 166) OR (95% CI)	Addiction (*n* = 154) OR (95% CI)	*p*-Value
BSQ-16				
No concern with shape	Crude OR	1.00 (ref.)	0.52 (0.34–0.82)	0.005
	Adjusted OR	1.00 (ref.)	0.48 (0.30–0.77)	0.002
Mild concern with shape	Crude OR	1.00 (ref.)	0.91 (0.54–1.56)	0.743
	Adjusted OR	1.00 (ref.)	0.94 (0.54–1.64)	0.830
Moderate and marked concern with shape	Crude OR	1.00 (ref.)	2.82 (1.61–4.91)	<0.001
	Adjusted OR	1.00 (ref.)	3.01 (1.70–5.33)	<0.001
Weekly Leisure-Time Activity				
Insufficiently Active/Sedentary	Crude OR	1.00 (ref.)	0.82 (0.52–1.29)	0.393
	Adjusted OR	1.00 (ref.)	0.88 (0.55–1.39)	0.576
Moderately Active	Crude OR	1.00 (ref.)	1.32 (0.73–2.39)	0.368
	Adjusted OR	1.00 (ref.)	1.40 (0.76–2.58)	0.288
Active	Crude OR	1.00 (ref.)	1.04 (0.67–1.61)	0.868
	Adjusted OR	1.00 (ref.)	0.95 (0.60–1.49)	0.818
WHO-5				
Worst well-being	Crude OR	1.00 (ref.)	3.32 (0.66–16.73)	0.145
	Adjusted OR	1.00 (ref.)	2.68 (0.53–13.64)	0.235
Poor well-being	Crude OR	1.00 (ref.)	1.37 (0.85–2.22)	0.197
	Adjusted OR	1.00 (ref.)	1.45 (0.88–2.37)	0.143
Mild well-being	Crude OR	1.00 (ref.)	0.97 (0.62–1.52)	0.884
	Adjusted OR	1.00 (ref.)	0.95 (0.60–1.49)	0.807
Good and best well-being	Crude OR	1.00 (ref.)	0.66 (0.40–1.07)	0.093
	Adjusted OR	1.00 (ref.)	0.66 (0.40–1.08)	0.098

Values are OR (95% CI). Abbreviations: BSQ-16: Body Shape Questionnaire 16-Item Version; CI: confidence interval; OR: odds ratio; adjusted OR: odds ratio adjusted by sociodemographic characteristics (age, gender, university student); WHO-5: World Health Organization-5 Well-being Index.

**Table 5:** Association between eating behaviors and social media addiction.

		No Addiction (*n* = 166) OR (95% CI)	Addiction (*n* = 154) OR (95% CI)	*p*-Value
Adult Eating Behavior Questionnaire (AEBQ)				
Enjoyment of food (EF)	Crude OR	1.00 (ref.)	1.33 (0.85–2.07)	0.209
	Adjusted OR	1.00 (ref.)	1.25 (0.79–1.97)	0.343
Emotional overeating (EOE)	Crude OR	1.00 (ref.)	2.24 (1.44–3.51)	<0.001
	Adjusted OR	1.00 (ref.)	2.25 (1.42–3.54)	0.001
Emotional undereating (EUE)	Crude OR	1.00 (ref.)	0.76 (0.49–1.19)	0.230
	Adjusted OR	1.00 (ref.)	0.73 (0.46–1.15)	0.176
Food fussiness (FF)	Crude OR	1.00 (ref.)	1.10 (0.70–1.73)	0.672
	Adjusted OR	1.00 (ref.)	1.09 (0.69–1.72)	0.710
Food responsiveness (FR)	Crude OR	1.00 (ref.)	2.31 (1.47–3.61)	<0.001
	Adjusted OR	1.00 (ref.)	2.13 (1.35–3.36)	0.001
Hunger (H)	Crude OR	1.00 (ref.)	2.18 (1.39–3.41)	0.001
	Adjusted OR	1.00 (ref.)	2.16 (1.37–3.41)	0.001
Slowness in eating (SE)	Crude OR	1.00 (ref.)	1.02 (0.66–1.58)	0.934
	Adjusted OR	1.00 (ref.)	0.99 (0.63–1.56)	0.954
Satiety responsiveness (SR)	Crude OR	1.00 (ref.)	1.48 (0.93–2.35)	0.097
	Adjusted OR	1.00 (ref.)	1.47 (0.91–2.37)	0.118

Values are OR (95% CI). Abbreviations: CI: confidence interval; OR: odds ratio; adjusted OR: odds ratio adjusted by sociodemographic characteristics (age, gender, university student).

## Data Availability

There are restrictions on the availability of the data in the research, due to the signed consent agreements around data sharing, which only allow access to external researchers for studies following the project purposes. Requestors wishing to access the data used in this study can make a request to the last author: cristina.bouzas@uib.es. The request will then be passed to the authors for deliberation.

## Abbreviations

AEBQ: Adult Eating Behavior Questionnaire; BSMAS: Bergen social media usage scale; BSQ-16: Body Shape Questionnaire 16-Item Version; IQR: interquartile range; OR: odds ratio; WHO-5: World Health Organization-5 Well-being Index.
